# Investigating the perception dimensions of sports disciplines stigma in China: social identity and participation willingness

**DOI:** 10.3389/fpsyg.2025.1476138

**Published:** 2025-04-28

**Authors:** Mengkai Wang, Yongtao Zhang, Mingtao Wang, Xi Zhao

**Affiliations:** School of Economics and Management, Chengdu Sport University, Chengdu, China

**Keywords:** stigma in sports, perception dimensions, participation willingness, mixed methods research, society identity

## Abstract

**Introduction:**

This study aims to explore the dimensions of stigma perception in sports disciplines, focusing on the impact these dimensions have on the public’s willingness to participate in stigmatized sports. The research seeks to identify and analyze the dimensions of stigma, test hypotheses regarding their influence, and investigate the mediating role of social identity in this relationship.

**Methods:**

The study was conducted in China using in-depth interviews with 36 sports enthusiasts, followed by a comprehensive questionnaire survey. Grounded theory was employed for the qualitative analysis of interview data, resulting in the identification of three primary dimensions of stigma: participant group stigma, negative events stigma, and discipline value stigma. Structural Equation Modeling (SEM) and path analysis were utilized to validate the proposed model and analyze the mediating effects.

**Results:**

We find that all three dimensions significantly influence the willingness to participate in sports disciplines, with negative events stigma having the most substantial impact. Social identity partially mediates the effects of these stigma dimensions on participation willingness, indicating that the perceived stigmas negatively impact social identity and participation willingness.

**Discussion:**

The results underscore the importance of addressing stigma in promoting sports participation. The findings align with existing literature on the negative impacts of stigmatization but extend the understanding by highlighting the mediating role of social identity. The study suggests that efforts to reduce stigma and enhance social identity could significantly boost public engagement in sports. Future research should explore additional mediating variables and the long-term effects of stigma reduction interventions.

## Introduction

The dissemination of sports disciplines is often considered to have a strong positive correlation with the process of globalization. The wave of economic globalization continues to drive the globalization of the sports industry, fostering international partnerships, collaboration between sports organizations, and the global circulation of sports resources (Liu et al., 2017; Major and O'Brien, 2005). Against this background, many emerging sports disciplines are entering China. Currently, under the influence of the two major policy strategies of national fitness and Healthy China, the focus of China’s sports development is shifting from highlighting the number of gold medals representing competitive sports levels to the overall development level of social sports participation representing the entire society (Zhang and Saunders, 2020). In this process, the socialization and marketization of Chinese sports are also accelerating, and the public has higher demands for the quantity and quality of sports. How to promote the development of various sports programs, expand the population engaged in regular physical exercise, and better serve the national fitness initiative has become a key task in current sports work in China.

However, in China, stigmatization of sports disciplines occurs frequently. The stigmatization of sports disciplines refers to the high degree of negative and difficult-to-eliminate stereotypes that the public holds about a particular sports activity. These stereotypes have rapid and widespread dissemination characteristics. For example, the sport of Ultimate Frisbee has been labeled with derogatory terms such as “frisbee girl” and “mating call,” while golf has been stigmatized as an “official’s sport” (Song et al., 2022; Zhao and Zhu, 2021). Such labels lead these sports to become stigmatized disciplines. Due to courtesy stigma, those associated with or related to the stigmatized entities also suffer devaluation, inevitably becoming affected by the stigma (Ali et al., 2012; Liu and Kozinets, 2022). In the context of stigmatized sports disciplines, participants, who are directly associated with these activities, are inevitably influenced by courtesy stigma. They are compelled to adopt more conservative self-management measures, such as avoiding participation in stigmatized sports and reducing their exposure related to these activities, to maintain their identity within the broader social circles and mitigate the stigma’s impact on themselves. This situation results in a vicious cycle of negative development for the stigmatized sports disciplines.

The core issue of sports disciplines stigma and the resulting derivative stigma phenomena not only hinder the promotion of the stigmatized sports but also severely impact the development of their related industries. This negative influence extends beyond the realm of sports, significantly affecting the implementation of national fitness strategies and the achievement of high-quality development goals in the sports industry. Therefore, Exploring the structural dimensions of sports stigmatization from a psychological perspective, understanding its formation mechanisms and impacts, and subsequently finding effective countermeasures holds significant theoretical and practical value.

Based on the above discussion, this paper aims to focus on exploring the current structural dimensions of sports disciplines stigma. We seek to develop a comprehensive measurement scale grounded in these structural dimensions and to examine the multifaceted impacts of each dimension on sports disciplines participation.

## Literature review

### The advancements in stigma theory and its application

The term ‘stigma’ originates from ancient Greece, where individuals, such as slaves, criminals, and traitors, were marked with symbols as a means of labeling and banishment. This act of marking constituted what is now referred to as stigma. Since Goffman’s seminal work in 1963, conceptualizing stigma as an inherently unreliable attribute that transforms an individual from a complete, ordinary person to one with a blemished character (Goffman, 1963) the definition of stigma has undergone several developments and supplements (Link and Phelan, 2001). Stafford and Scott (1986) and others have proposed that stigma involves characteristics opposite to societal norms. Drawing on Goffman’s definition, the concept of discrimination is subsequently introduced to refine the understanding of stigma, which is delineated as the ascription of undesirable traits to individuals. Link and Phelan (2001), in consolidating previous research, constructed modified concepts related to stigma, emphasizing its expansion into various facets of social life with societal development. Therefore, a multidimensional sociological exploration of the nature and consequences of stigma becomes crucial from a multidimensional sociological perspective.

Presently, Ongoing scholarly debates persist on the precise definition and mechanisms of stigma. Rooted in social psychology, ‘stigma’ is comprehended as personal attributes or labels deemed societal defects (Major and O'Brien, 2005). Individuals subjected to stigma are perceived as lacking certain normal attributes (Pescosolido and Martin, 2015). The formation mechanism of stigma involves public stigma, which impacts individuals through enacted stigma, perceived stigma, and internalized stigma, leading to self-stigmatization (Bos et al., 2013).

As stigma theory permeates various disciplines, marketing has witnessed corresponding research. In marketing, stigma is defined as negative labels, biases, and derogatory attributes associated with interest groups and their products or services for commercial purposes (Mirabito et al., 2016). Scholars, with a focus on consumer behavior, have delved into the impact of stigmatized products on consumer shopping behavior. For instance, Shin and Jin (2021) investigated the willingness of consumers, driven by social status pursuit, to purchase fur products amid increasing global animal protection awareness. Barlow et al. (2016) explored the influence of stigmatized products on the industry category, using craft beer in the United States as an example. Results consistently showed that both consumer group and product stigmatization significantly negatively affect shopping behavior.

Brands, like products, face stigmatization. Certain brand attributes can create impressions that may lead to brand stigma, such as the brand’s country of origin (Wang et al., 2004), brand category, or brand fit (Lafferty, 2007). In the current landscape, scholars propose that the resolution of industry stigma relies heavily on corporate social responsibility (CSR) tools, involving sponsorship or charitable actions to improve the tainted image of the industry (Palazzo and Richter, 2005).

### Stigmatization studies in sport

Existing research on sports stigmatization predominantly concentrates on specific disciplines, groups, and brands, with a noticeable dearth of macro-level studies on the perception dimension of stigmatization in sports.

The examination of sports participation group stigmatization has been undertaken. Lee et al. (2021) employed attribution theory to investigate the mechanisms underpinning the formation of athlete group stigma and analyzed its resultant impacts. The study found that internal attribution, in comparison to external attribution, intensifies public stigma towards athletes, negatively affecting the willingness to purchase products associated with stigmatized athletes. Teachers, especially in physical education, face biases rooted in stereotypes, with sports teachers often associated with aggression (McCullick et al., 2010). Traditionally, physical education has been associated with ideals of toughness, strength, and competitiveness (White and Hobson, 2017). Under this paradigm, male physical education teachers are sometimes unfairly characterized as overly aggressive or unrefined in their leadership, while female physical education teachers may be stigmatized as exhibiting masculine traits—often labeled as tomboys or even as homosexual. These negative stereotypes have been perpetuated and amplified by portrayals in film and television over time (McCullick et al., 2010; Walton-Fisette et al., 2017).

In addition to sports participation group stigmatization, published studies also research on the stigmatization of specific sports disciplines. Taking disc golf as an example, media and reports often portray it as a non-competitive and non-intensive sport, contributing to its stigmatization (McGinnis et al., 2016). Despite efforts to reduce land use and environmental impact, disc golf is still labeled by environmental organizations as a damaging activity, resulting in defamation and stigma (McCullick et al., 2010). There are also studies focusing on the stigma experienced by professional cyclists within the context of clean sports. The excessive use of banned substances in past competitions, coupled with inadequate regulation, generated negative impressions, leading to stigma associated with professional cycling and its athletes. The study discussed and summarized the coping measures of professional cyclists under stigma, categorized into self-restraint, strengthened supervision, and social advocacy stigma resolution chain (Sefiha, 2017).

Concerning sports brand stigmatization, brand image, as a crucial intangible asset (Aaker and Keller, 1990), serves as a comprehensive evaluation and reflection of consumer perceptions of product characteristics and the overall spirit of the enterprise. Brand stigmatization occurs when consumers impose negative labels on a brand, damaging its image and reputation. Gerke et al. (2014) focusing on the country of origin’s impression, used outdoor sports brands from New Zealand as an example, demonstrating that consumer perceptions transfer specific impressions of the brand’s home country to the sports brand and its products. The higher the consistency between brand products and the consumer’s perception of the brand’s home country, the greater the consumer’s approval of the sports brand and its products, influencing purchasing behavior.

Finally, stigmatization related to group attributes involves gender, sexual orientation, body shape, and social status. These factors may incur varying degrees of stigma, impacting sports participation. In the realm of sports, diversity efforts are primarily propelled by commercial interests rather than a social justice framework (Hartmann-Tews, 2023). Consequently, sexual minority groups such as ‘LGBTQ+’ lack commercial interests, leading to negative societal and market responses and exacerbating the unfair treatment of sexual minority groups in sports (Baeth et al., 2023; Denison et al., 2021). Thedinga et al. (2021) asserted that individuals with obesity often face shame and discrimination related to weight in sports and exercise environments, leading them to cope with these experiences by excluding themselves from sports and exercise.

### Research on factors influencing the choice of sports disciplines

Scholars have investigated various factors influencing the choice of sports disciplines at different levels, broadly categorized as intrinsic and extrinsic factors.

Starting from intrinsic factors, Casper et al. (2011) constructed a model based on ecological system theory, highlighted the impact of participants’ preferences, personalities, and habits, in addition to external factors from school and family, on their sports participation and disciplines selection. Bevan et al. (2022) identified social psychological factors, such as anxiety about physique and appearance, as influencing individuals’ concepts and choices in sports participation, which exacerbate self-stigmatization and hinder their love for and involvement in sports (Somerset and Hoare, 2018).

The selection of sports activities by individuals is also influenced by external factors, such as the life stage of the participants and societal evaluations of the particular sport (Kamphuis et al., 2008; Pot et al., 2014). These external elements play a significant role in shaping people’s choices in sports participation. Jenkin et al. (2017) found that the selection of sports disciplines by the elderly is influenced by factors such as the practicality of sports disciplines, health benefits, and participation costs. Additionally, the gender ratio within the school can also influence students’ choices and participation in sports disciplines. Gender imbalance may lead schools to prioritize providing sports resources for the gender-dominant group, thereby influencing students’ choices and involvement in sports disciplines (Ferry and Lund, 2018).

### Literature summary

In summary, stigmatization is a multifaceted structure encompassing perceptual, categorical, and reactive dimensions. When a particular sport is stigmatized, it weakens the public’s perception of the sport, causing certain sports participation groups to fall into a ‘self-stigmatization’ trap. This impedes the socialization process of the sports disciplines and subsequently affects the development of the disciplines associated industries. Reflecting on past literature, this paper observes a concentration on sports teachers, specific sports disciplines, sports brands, and stigmatized groups. The emphasis on pathways to resolve stigma is evident, but a systematic study of the perception dimensions of stigmatization in sports disciplines at a macro level is lacking. Therefore, this paper adopts a mixed research method, presenting two research objectives. The first, grounded in grounded theory, aims to elucidate the perceptual dimensions of stigmatization in sports disciplines. The second, through questionnaire data collection, utilizes structural equation modeling to analyze the influence of various dimensions on the degree of perceived stigma in sports disciplines. The conclusions drawn from this study will broaden the research boundaries of stigmatization in sports disciplines and provide theoretical guidance for the positive development of sports disciplines.

## Study 1: analysis of sports disciplines stigmatization dimensions based on grounded theory

### Methods

Grounded theory referred to the adoption of a bottom-up research approach, employing a systematic inductive analysis procedure. Based on empirical data, it sought to identify core concepts relevant to the research problem, thereby constructing a rigorous and scientific theory (Charmaz, 2014). Procedural grounded theory emphasized human subjective understanding and highlighted the interconnection of existing experiences and hypothetical theories through causal relationships. This paper utilized the three-level coding of procedural grounded theory to analyze textual data obtained through in-depth interviews.

### Data collection

Grounded theory research required the use of purposive sampling in interviews to enhance result diversity (Gratton and Jones, 2004). Consequently, this study employed purposive sampling with specific inclusion criteria. Interviewees were selected based on their participation in or observation of at least one sports discipline within the past year and their familiarity with public perceptions or stereotypes surrounding certain sports. To ensure a diverse range of perspectives, potential participants were identified via established sports networks, online forums, university clubs, and social media groups. The research team directly contacted these individuals via email and messaging platforms, provided a detailed explanation of the study’s objectives, and obtained informed consent prior to conducting the interviews.

Strict adherence to ethical standards was maintained throughout the process—from the selection of interview subjects to the interview process and subsequent data handling—to ensure that participants’ personal privacy was fully protected. The interviews focused on three main aspects: (a) interview explanations, (b) basic information about interviewees, and (c) semi-structured interview questions, as outlined in Table 1. Informed consent was obtained from all participants, who were fully briefed on the purpose and content of the interviews, and participation was entirely voluntary. Participants were also assured of the confidentiality and privacy of their responses.

**Table 1:** Semi-structured interview outline.

Serial number	Basic questions
1	What considerations guide your participation in a specific sporting activity?
2	In your estimation, which sporting endeavors are notably influenced by public prejudice?
3	Please provide an analysis of the factors contributing to bias against these sports, as per your understanding.
4	Are there sports in your everyday life that elicit feelings of aversion or discrimination?
5	Kindly elucidate on the specific aspects that prompt your disapproval, and the discriminatory nature associated with these sports.
6	Have you encountered instances of bias or discriminatory conduct while engaged in a particular sport?
7	(If affirmative) Please specify the manifestations of these biases or discriminatory behaviors.

The interviews yielded a total of 36 interviewees. The data from 26 interviewees were transcribed into text, resulting in approximately 82,000 words of textual data. The procedural grounded theory method was employed to sequentially analyze the textual data, generating initial concepts, subcategories, and main categories, ultimately forming the dimensions of stigma perception in sports disciplines. The data from the remaining 10 interviewees were used to test theoretical saturation.

### Open coding

Open coding involved conceptualizing and categorizing acquired data by gradually organizing raw data based on specific principles. This process utilized concepts and categories to accurately represent the data’s content, resulting in the inductive summarization of abstract concepts. To streamline the analysis and minimize interference from complex textual data, the raw interview transcripts (82,000 words from 36 interviews) were rigorously cleaned by removing habitual phrases (e.g., colloquial fillers), irrelevant sentences, and content misaligned with interviewees’ original intentions. For instance, statements like “Discrimination is encountered when my technical skills in the sport are insufficient” were stripped of non-essential language and retained as core excerpts. Subsequently, the refined data underwent preliminary conceptualization. Through line-by-line analysis, 28 initial concepts were inductively extracted. Examples include: technical discrimination (e.g., exclusion due to skill level) and social motivation (e.g., participating to network with high-status individuals). These concepts were iteratively compared, refined, and grouped into 10 subcategories. Redundant or overlapping concepts (e.g., gender bias) were merged to ensure clarity. Table 2 illustrates this process, mapping interview excerpts to concepts (e.g., “body-shape discrimination” under Group Participation Behavior) and their evaluative properties (e.g., “good/bad”).

**Table 2:** Examples of open coding.

Interview content coding	Conceptualization	Categorization	Category Properties	Evaluative Properties
Discrimination is encountered when my technical skills in the sport are insufficient. Feeling excluded as a result of perceived shortcomings in my physique. Some individuals argue against the involvement of females in XX sports …	Technical discrimination Body-shape discrimination Gender discrimination …	a1 Group Participation Behavior	Behavior exhibited when groups participate in sports disciplines	Good/bad
More for taking photos, and showing off. They participate in this sport merely to get to know people of social standing. …	Showing off motivation Social motivation …	a2 Group Participation Motivation	Motivation and objectives pursued by the group participating in a specific sports activity	Good/bad
Always wearing clothing unsuitable for sports These sports are meant for the participation of wealthy individuals. .…	Clothing image Social image …	a3 Group Participation Image	Appearance and Inner Image of Participating Groups	Appropriate/ Inappropriate
Many athletes in XX sports abuse performance-enhancing drugs Intentional interference with opponents is common in ice sports. The widespread existence of gambling is undermining the public’s impression of XX sport. …	Drug violations. Contest violations Betting on contest …	a4 Contest Violations and Prohibitions	Violations and prohibitions arising around a certain sports disciplines	More/less
Occupation of our football fields by those who play xx sports The ordinary sport has been co-opted as a means of socializing and matchmaking. The space needed for XX sport could be used to build multiple basketball courts …	Occupation of the space Value displacement Waste of resources …	a5 Encroachment of Social Resources	Sports disciplines’ occupation and waste of social resources	More/less
Match-fixing and biased refereeing are prevalent in xx sports Incidents of fan riots are common in xx sports …	Corruption in competitions Fans riots …	a6 Deviant Violations	Unethical and illegal behavior surrounding a certain sport	More/less
Videos of XX sport often exploit the appeal of female participants by focusing on their appearance and attire. There are often numerous rumors surrounding well-known athletes, but the majority of these are mostly untrue. …	Malicious reporting Distortion of Facts …	a7 Malicious Media Coverage	Negative and false publicity related to sports disciplines	More/less
After watching a match of XX, it felt overwhelmingly dull. XX sport lacks competitiveness and is only played by individuals with poor physical fitness. XX sport involves a higher risk of injuries due …	Sporting Attractiveness Sporting competitiveness Sporting risks …	a8 Sporting Participatory Value	Attraction of the Sports Disciplines in Terms of Spectatorship and Participation	High/Low
There are hardly any competitions for XX sports in China The xx sport lacks high-level leagues. …	Number of events Level of events …	a9 Sporting Competitive Value	Competitive Attraction Value of the Sports Disciplines	High/Low
The XX sport has lost many of its original movement techniques over the years. In my impression, XX sport is often associated with playing with pets I dislike these emerging trendy sports …	Cultural misalignment Cultural absence Subcultural identification …	a10 Sporting Culture Value	Cultural Value of the Sports Disciplines	High/Low

Open coding involves extensive text analysis. To save space, this table presents only a partial display.

This study primarily used the expression ‘alphabet letter + number’ to code categories for subsequent analysis. In summary, the 10 subcategories finally organized were: Group Participation Behavior (a1), Group Participation Motivation (a2), Group Participation Image (a3), Contest Violations and Prohibitions (a4), Encroachment of Social Resources (a5), Deviant Offenses (a6), Negative Media Coverage (a7), Sporting Participatory Value (a8), Sporting Competitive Value (a9), Sporting Culture Value (a10).

### Axial coding

Axial coding was the second stage of the coding process, involving continuous comparison and classification. It aimed to connect primary and secondary conceptual categories into a relational network. Based on this, this study synthesized the 10 subcategories into relational main categories through constant comparative analysis, this hierarchical aggregation resolved overlaps (merging Deviant Offenses and Contest Violations under Negative Events Stigma), resulting in three main categories: stigma of participant groups, stigma of discipline value, and stigma of negative events (see in Table 3).

**Table 3:** Main results for axial coding and selective coding.

Core categories	Main categories	Corresponding categories	Encompassed concepts
Sports disciplines stigma-aware dimensions	Participation group stigma	al Group participation behavior a2 Group participation motivation a3 Group participation image	Technical discrimination; Body-shape discrimination; Gender discrimination (3 concepts) Showing off motivation; Social motivation (2 concepts) Clothing image; Social image; Sexual orientation (3 concepts)
	Negative events stigma	a4 Contest violations and prohibitions a5 Encroachment of social resources a6 Deviant violations a7 Malicious media coverage	Drug violations; Contest violations; Betting on contest (3 concepts) Occupation of the space; Value displacement; Waste of resources (3 concepts) Corruption in competitions; Fans riots (2 concepts) Malicious reporting; Distortion of Facts (2 concepts)
	Disciplines value stigma	a8 Sporting participatory value a9 Sporting competitive value a10 Sporting cultural value	Spectatorship; Competitiveness; Safety (3 concepts) Event types; Number of events; Event popularity; Event professionalism (4 concepts) Cultural absence; Subcultural identity; Cultural dislocation (3 concepts)

### Selective coding

Selective coding referred to the process of choosing core concepts within the identified categories. Through continuous analysis, secondary concepts related to the core categories were concentrated, systematically explained, and the relationships between the main and secondary concepts were verified. Ultimately, the core category extracted was ‘Perception of Stigma in Sports.’ Based on this, the paper identified three dimensions of stigma perception in sports: stigma associated with participant groups, stigma related to negative events, and stigma related to the value of the sports disciplines.

### Theoretical saturation testing

The saturation test of the theory examined whether the concepts and categories proposed at each level of coding were sufficient. It involved progressively abstracting, summarizing, and inducing from the initial data. If the relevant codes did not generate new concepts, it indicated that the theoretical model was relatively saturated and passed the saturation test. In this study, coding analysis was performed on the remaining 10 transcripts of in-depth interviews. The results showed that the latest coded concepts could be entirely incorporated into existing concept codes without generating new concepts and categories. Therefore, it could be considered that the theoretical model had achieved saturation.

## Study 2: the impact of stigmatization perception dimensions on the degree of stigmatization in sports disciplines

In Study 1, through conversations with respondents, we found that some respondents did not immediately reduce their willingness to participate in stigmatized sports after perceiving the stigma. Instead, they experienced a process of concern. For example, one respondent stated, “Many people think that participating in ultimate frisbee, especially for women, is a way to meet the opposite sex. If I continue to participate and share it online, I am worried about being misunderstood (Respondent 6, female).” We hypothesize that perceived stigma triggers public concern about social identity associated with continued participation in such sports, leading to a decreased willingness to participate.

Social identity, as an interactive social psychological theory, investigates the role of self-concept, cognitive processes, and social beliefs in group processes and intergroup relations (Hogg, 2016). It emphasizes key concepts such as self-concept, social identity, and group processes. Based on speculations drawn from existing research (Rees et al., 2015), in the context of sports as a human-centric endeavor, the stigma attached to specific sports disciplines is inevitably expected to impact participants. Awareness of cultural negative stereotypes surrounding certain sports disciplines, such as being overweight, makes individuals vulnerable to social identity threats triggered by concerns about potential devaluation, discrimination, rejection, or negative stereotyping (Hunger et al., 2015). This influence not only creates an identity barrier between groups participating in stigmatized sports and those in conventional sports but also extends to affect the group’s sense of identification and belongingness towards the stigmatized sports. Ultimately, these influences significantly shape the willingness of individuals to engage in stigmatized sports activities.

**Figure 1:**
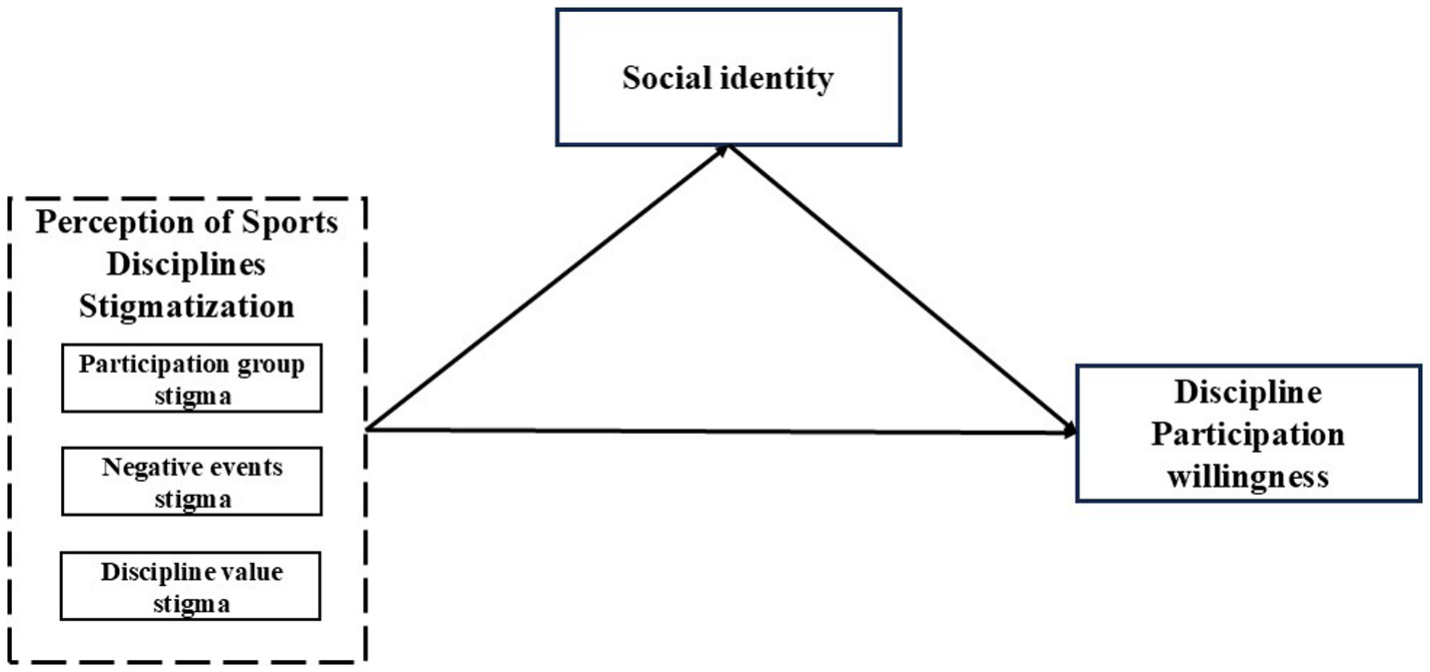
Standardized model of Study 2.

On this basis, we speculated that social identity plays a mediating role in the influence path of stigma perception of sports events on the willingness to participate in sports events. Therefore, we introduced social identity as a mediating variable and constructed a standardized structural equation model for further measurement (see in Figure 1).

### Study design

In study 2, we employed online survey method, utilizing the ‘Wenjuanxing’ platform for distribution and preliminary data processing. Wenjuanxing platform, a secure and widely used tool for academic research in China. The platform ensures participant anonymity, encrypts data transmission, and provides automated filtering of invalid responses (e.g., duplicate IP addresses or inconsistent answers to attention-check questions).To ensure the authenticity and validity of the data, two control questions were included in the questionnaire to filter out invalid responses. A total of 450 questionnaires were distributed, and after excluding those with incorrect answers to the control questions, 363 valid responses were retained, resulting in an effective response rate of 81%. The study adhered strictly to ethical standards, informing participants about the guidelines and purpose of the questionnaire after obtaining their consent.

## Results

### Reliability and validity analysis

SPSS 26.0 was employed to perform a reliability analysis on the scale. The results revealed an internal consistency reliability of 0.713 for the overall scale. For Participant group stigma, Negative events stigma, Disciplines value stigma, Social identity, and Disciplines participation willingness, the internal consistency reliabilities were 0.936, 0.931, 0.925, 0.935, and 0.963, respectively. These results affirm the reliability and stability of the constructed scale for the perception dimensions of sports disciplines stigmatization (see in Table 4).

**Table 4:** Reliability test results of the revised scale.

Latent Variable	Latent Variable	Cronbach’s α
Participation group stigma	6	0.936
Negative events stigma	5	0.931
Disciplines value stigma	5	0.925
Social identity	5	0.935
Disciplines participation willingness	3	0.963

### Exploratory factor analysis

After confirming the reliability of the scale, we conducted an exploratory factor analysis (EFA). First, we performed the Kaiser-Meyer-Olkin (KMO) test and Bartlett’s test of sphericity on the scale items. A KMO value greater than 0.7 and a Bartlett’s test *p*-value less than 0.01 indicate a high level of reliability for the scale (see in Table 5), allowing us to proceed with the factor analysis.

**Table 5:** Results of KMO and Bartlett spherical test.

KMO and Bartlett test		Value
KMO value		0.953
Bartlett	Approximate chi-square	8350.932
	df	276
	*p*	<0.001

Using principal component analysis and the varimax rotation method, we extracted factors from the 24 items based on eigenvalues greater than 1. Five common factors were extracted from the 24 items, with a cumulative variance contribution rate of 80.015%, which exceeds the 60% threshold. This indicates that the five factors explain 80.015% of the variance of the 24 items. The detailed results of the exploratory factor analysis are presented in Table 6.

**Table 6:** Scale items, sources, and standardized regression weights.

Dimensions	Items	Standardized regression weights	Standard Error	Source
Participation group stigma	I do not appreciate the appearance presentation of the xx sports community.	0.874	0.047	In-depth Interview (Sartore and Cunningham, 2010)
	The clothing image of the xx sports community does not conform to the general societal norms.	0.857	0.049	
	I perceive an arrogant and aloof attitude from the xx sports community.	0.856	0.047	
	I think that xx sports are more suitable for specific gender participation	0.800	0.056	
	The overrepresentation of sexual minority groups in xx sports makes me uncomfortable	0.834	0.053	
	I believe the motives of the xx sports participants are more for show	0.835		
Negative events stigma	I believe the xx sport has a higher frequency of violent incidents and deviant behavior	0.835	0.046	In-depth Interview (Goepfert et al., 2019)
	I think that xx sports further propagate body image anxiety	0.876	0.046	
	I think that xx sports intensify controversies regarding gender topics	0.843	0.048	
	I think that xx sports are considered a hotspot for competition corruption	0.854	0.043	
	In the promotional process, xx sports deliberately use and amplify their own stigma	0.865		
Disciplines value stigma	I think that the engagement experience of xx sports is low	0.786	0.048	In-depth Interview (Pavlidis and Fullagar, 2012)
	I think that the viewing experience of xx sports is low	0.874	0.049	
	I think that the competitive elements of xx sports is low	0.870	0.051	
	I believe the culture represented by the xx sport is niche and rebellious	0.855	0.050	
	I believe the xx sport poses a threat to mainstream sports culture	0.837		
Social identity	When someone praises this sport, it feels like a personal compliment.	0.875		In-depth Interview (Mael, 1988)
	I am very interested in what others think about the sport.	0.892	0.049	
	When someone criticizes this sport, it feels like a personal insult	0.854	0.049	
	When I talk about this sport, I usually say ‘we’ rather than ‘they	0.859	0.046	
	If a story in the media criticized this sport, I would feel embarrassed	0.837	0.050	
Disciplines participation willingness	Stigma will make me lose interest in this sport	0.962		In-depth Interview (Ganesh et al., 2000)
	Stigma will deepen my negative impression of this sport	0.956	0.024	
	Stigma will reduce my willingness to participate in this sport	0.925	0.027	

### Descriptive statistics and correlation analysis

The results indicated that the three dimensions of perceived stigma in sports were significantly negatively correlated with both the willingness to participate in the sport and social identity. At the same time, social identity was significantly positively correlated with the willingness to participate in the sport. Additionally, the three dimensions of perceived sports stigma were significantly positively correlated with each other (see in Table 7).

**Table 7:** Results of descriptive statistics and correlation analysis.

	M ± SD	1	2	3	4	5
1. PGS	3.61 ± 1.34	1				
2. NES	3.71 ± 1.24	0.644**	1			
3. DVS	3.59 ± 1.23	0.606**	0.617**	1		
4. SI	3.75 ± 1.14	−0.407**	−0.396**	−0.415**	1	
5. PW	3.99 ± 1.75	−0.684**	−0.701**	−0.684**	0.562**	1

Significant at the 0.01 level (two-tailed). PGS refer to Participation group stigma; NES refer to Negative events stigma; DVS refer to Disciplines value stigma; SI refer to Social identity; PW refer to Participation willingness.

### The model fit test

Confirmatory factor analysis results showed: χ2 = 389.451, df = 242, χ2/df = 1.609, RMSEA = 0.041, GFI = 0.919, CFI = 0.982, IFI = 0.982, TLI = 0.980. Following the criteria for judging model goodness-of-fit, the output results of confirmatory factor analysis were all within an acceptable range (see in Table 8). Hence, it was evident that the sports disciplines stigma scale had good structural validity.

**Table 8:** Model fit test results.

Fit index	$x^{2}/df$	RMSEA	GFI	CFI	IFI	TLI
Recommended value	<3	<0.8	>0.9	>0.9	>0.9	>0.9
Observed Value	1.609	0.041	0.919	0.982	0.982	0.980

### Main effects analysis

Subsequently, a structural equation model and path analysis were conducted. The results of the main effects analysis (see in Table 9) indicated significant negative effects of stigma associated with participant groups, negative events stigma, and disciplines value stigma on the willingness to participate in sports disciplines (*β*_Participant group stigma_ = −0.236, β _Negative events stigma_ = −0.303, β _Disciplines value stigma_ = −0.258). Considering the magnitude of the path coefficients, differences emerged in the impact of the three dimensions on the perception of stigma in sports disciplines. Stigma associated with negative events had the most substantial impact on willingness to participate, followed by disciplines value stigma, while participant group stigma had the smallest impact.

**Table 9:** Path analysis.

	Standardized Path Coefficient	Unstandardized Path Coefficient	Standard Error	t	P
Participation group stigma →Disciplines Participation willingness	−0.236	−0.313	0.067	−4.694	***
Negative events stigma → Disciplines Participation willingness	−0.303	−0.427	0.072	−5.949	***
Disciplines value stigma →Disciplines Participation willingness	−0.258	−0.372	0.070	−5.304	***

*** Represents *p* < 0.001.

### Mediation effects analysis

For the examination of mediation effects in the perception of stigma variables, the study employed the Bootstrap method using Amos Graphics with 5,000 resampling settings and a confidence level of 95%. To determine whether a variable has a mediation effect, it is crucial to observe whether the 95% confidence interval (95% CI) of the regression coefficient ab includes 0. If 0 is not included in the 95% CI, there is a mediation effect. If 0 is included in the 95% CI, there is no mediation effect. If both a, b, and c’ are significant, and the sign of ab is the same as c’, then a partial mediation effect is observed. If at least one of a and b is not significant, and the 95% CI of ab includes 0 (non-significant), then there is no significant mediation effect. According to Table 10, social identity partially mediates the impact of stigma associated with participant groups, negative events stigma, and disciplines value stigma on the public’s willingness to participate in sports disciplines.

**Table 10:** Results of mediating effect test.

Path	C total effect	*a*	*b*	ab mediating effect	ab (95%CI)	c’ direct effect	Conclusions
PGS → SI → PW	−0.375*	−0.191*	0.246**	−0.047*	[−0.131, −0.013]	−0.313*	Partial mediation effect
NES → SI → PW	−0.483*	−0.161*	0.246**	−0.040*	[−0.121, −0.004]	−0.427*	Partial mediation effect
DVS → SI → PW	−0.446*	−0.209*	0.246**	−0.051*	[−0.152, −0.018]	−0.372*	Partial mediation effect

* Indicates *p* < 0.05, ** indicates *p* < 0.01.

## Discussion

This study employed a mixed-methods approach to explore the stigma associated with sports disciplines in China. Our qualitative analysis revealed three key dimensions of stigma—participant group stigma, negative events stigma, and disciplines value stigma—each of which contributes uniquely to the public’s negative perceptions of certain sports. The subsequent quantitative phase confirmed that these dimensions significantly reduce the willingness to participate in sports, with negative events stigma showing the strongest influence (*β* = −0.303). Besides, our findings indicate that social identity partially mediates the relationship between these stigma dimensions and participation willingness, highlighting the role of social identity in shaping sports engagement.

Participant Group Stigma is a critical dimension, encompassing group motivation, characteristics, and social image, which significantly affect public participation willingness. Beyond physical appearance-related stigma (Bevan et al., 2022; Inderstrodt-Stephens and Acharya, 2017), this study highlights the role of gender and sexual orientation in stigma formation, emphasizing the motivation-behavior chain and enriching the understanding of stigmatization dimensions; Negative Events Stigma is the most impactful dimension, surpassing participant group and disciplines value stigma in its effect on public perception. It includes misconduct during events and negative media coverage, which significantly reduce participation willingness. For instance, football hooliganism has been linked to violence, damaging public identification with the sport (Frosdick and Marsh, 2013). The rapid dissemination of negative events through media further amplifies stigma (Hill and Fuller, 2018). This study extends the scope of negative events to include operational issues in sports organizations, which exacerbate public resentment and stigma perception; Disciplines Value Stigma represents a novel dimension, focusing on how deviations from sports norms and values contribute to stigmatization. High-risk sports, for example, are perceived as non-conforming and insecure, clashing with mainstream culture (Vanreusel and Renson, 1982; Wheaton, 2015). This subcultural value system reinforces stigma, shaping negative public opinions and highlighting the need to explore the cultural dynamics of high-risk sports.

Social Identity as a Mediator: Social identity plays a key role in linking disciplines value stigma, participant group stigma, and negative events stigma with the willingness to participate in sports. According to social identity theory, people form distinct social groups based on shared characteristics. Sports participation offers a venue where these groups interact and sometimes conflict, leading to the formation of exclusive networks that reinforce group identity while excluding outsiders. The rise of social media has further amplified this exclusivity, intensifying negative opinions and stereotypes that contribute to the overall stigma of sports disciplines.

## Conclusion

This study systematically identified three key dimensions of stigma in Chinese sports disciplines—participant group stigma, negative events stigma, and discipline value stigma—and demonstrated their significant negative impact on public participation willingness. Among these, negative events stigma exerted the strongest influence, underscoring the role of media and organizational misconduct in amplifying public aversion. Social identity emerged as a critical mediator, linking stigma perceptions to reduced engagement, thereby highlighting the psychological mechanisms underlying participation barriers. The findings advance theoretical frameworks on sports stigmatization by integrating multidimensional perspectives and emphasize the necessity of collaborative governance involving stakeholders to mitigate stigma.

### Implications

The stigmatization perception dimensions of sports disciplines, constructed in this study, reveal that the perception of disciplines stigmatization is a multidimensional variable. Unlike previous studies that focused on specific sports groups or individual disciplines, the stigmatization perception dimensions of sports disciplines aim to comprehensively explain the stigmatization factors of various sports disciplines at the conceptual level of ‘sports.’ Compared to previous research, which emphasized the external environment and public opinion for the mitigation of sports disciplines stigmatization, this study highlights the need for a shared and collaborative governance responsibility among multiple stakeholders. Only through such an approach can effective governance and mitigation of sports disciplines stigmatization be achieved.

Next, this study emphasizes that public perception of sports disciplines stigmatization is a complex dimension involving various aspects of society, government, groups, and individuals. Therefore, the improvement and alleviation of stigmatization in sports disciplines cannot solely rely on government initiatives or disciplines associations. It requires a multi-party shared responsibility and collaborative governance to efficiently advance the governance and mitigation efforts of sports disciplines stigmatization.

Ultimately, the stigmatization perception dimensions of sports disciplines, introduced from the public’s perspective, focus on exploring public awareness of stigmatized sports disciplines. We delve into the general perception dimensions of stigmatization in sports disciplines, offering a comprehensive interpretation of the stigmatization phenomena across various sports disciplines. From a theoretical perspective, this study provides an in-depth exploration of the sources of stigmatization perception dimensions in sports disciplines, moving beyond the study of specific disciplines or groups. In practical terms, the public’s perception of sports disciplines stigmatization is a complex dimension, and when faced with an already established stigmatization environment, the emphasis should not only be on the external environment and public opinion’s mitigation but also on the specific analysis of different sports disciplines. This includes seeking internal reforms and external environmental improvements as dual paths for the mitigation of stigmatization in sports disciplines.

At the same time, it is also important to emphasize the role of social identity in this process. Social identity not only serves as a transmitter of threats but also as a force for repair and reconciliation (Rees et al., 2015). A deeper understanding of the mediating role of social identity contributes to revealing the psychological mechanisms behind sports disciplines stigmatization and provides valuable insights for developing strategies to improve willingness to participate. This underscores the importance, in managing and promoting sports disciplines, of considering not only specific stigmatization factors but also individuals’ social identity and emotional aspects.

### Limitations and future research

This study mainly focused on the general public’s perspective when exploring the perception dimensions of sports disciplines stigmatization. In terms of the selection of interviews and questionnaires, the selection of interviews and questionnaires is mainly aimed at the public, and there is a lack of emphasis on professional groups such as professional athletes, sports administrators and policy makers. In the face of the current situation that some sports in China are seriously polluted and the causes are complex, future studies need to further expand the sample scope and further extend the level of stigma perception of sports in the study of professional sports groups, so as to obtain richer conclusions. Also, future studies might explore the dynamic interplay between public perceptions and those held within professional circles. Understanding how stigmatization influences and is influenced by different stakeholders can provide valuable insights for developing targeted interventions and strategies to mitigate the negative effects associated with stigmatized sports.

## Data Availability

The original contributions presented in the study are included in the article/supplementary material, further inquiries can be directed to the corresponding author/s.
